# Supplementary material for: High Permittivity Polymer Composites on the Basis of Long Single-Walled Carbon Nanotubes: The Role of the Nanotube Length
Source: Nanomaterials (Basel). 2022 Oct 10;12(19):3538. doi: 10.3390/nano12193538 (PMC9565907; doi:10.3390/nano12193538)
Supplement: Supplementary file 1 [file nanomaterials-12-03538-s001.zip › nanomaterials-1941180-supplementary.pdf]

Supporting Information

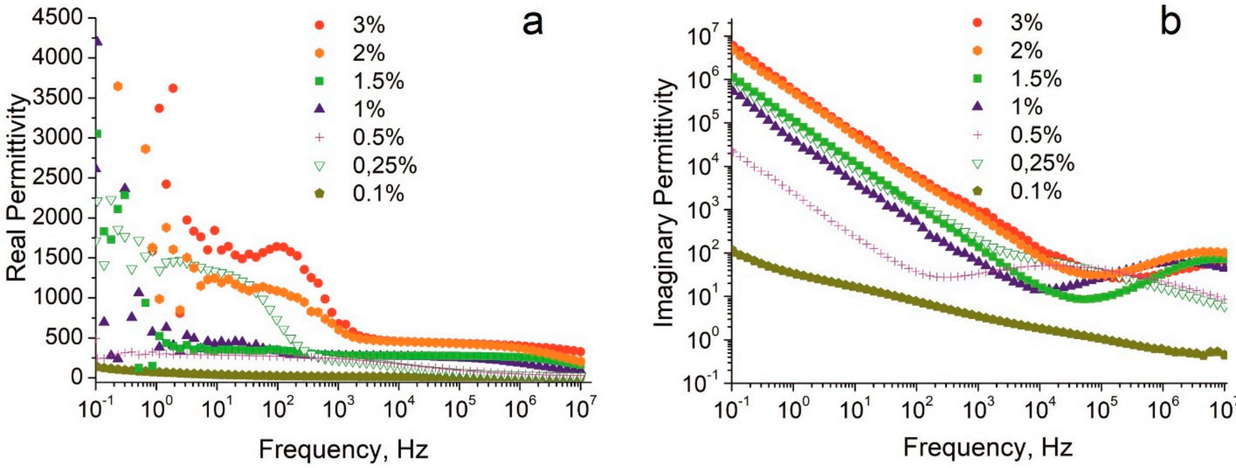

Figure S1. Frequency dependences of the real (a) and imaginary (b) parts of the complex permittivity for composites with different filler contents in the low-frequency region.

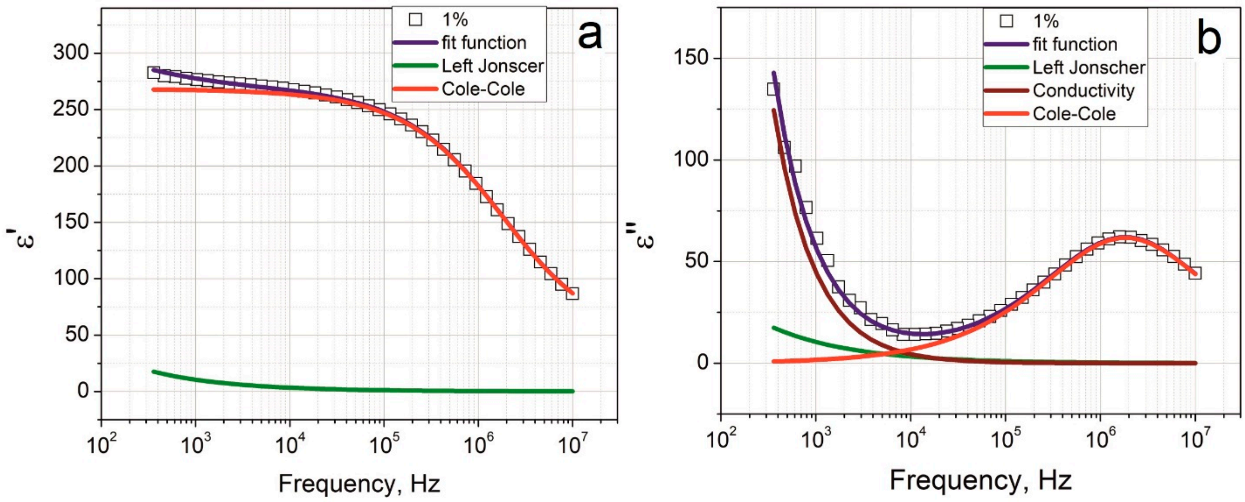

Figure S2. Approximation of the dielectric spectrum by the equation (1) for a sample with a filler content of 1%, (a) real part, and (b) imaginary part. The violet line is the fitting function; the green line is the Johnsher parameter; the brown line is DC-conductivity; the red line is the Cole-Cole function.

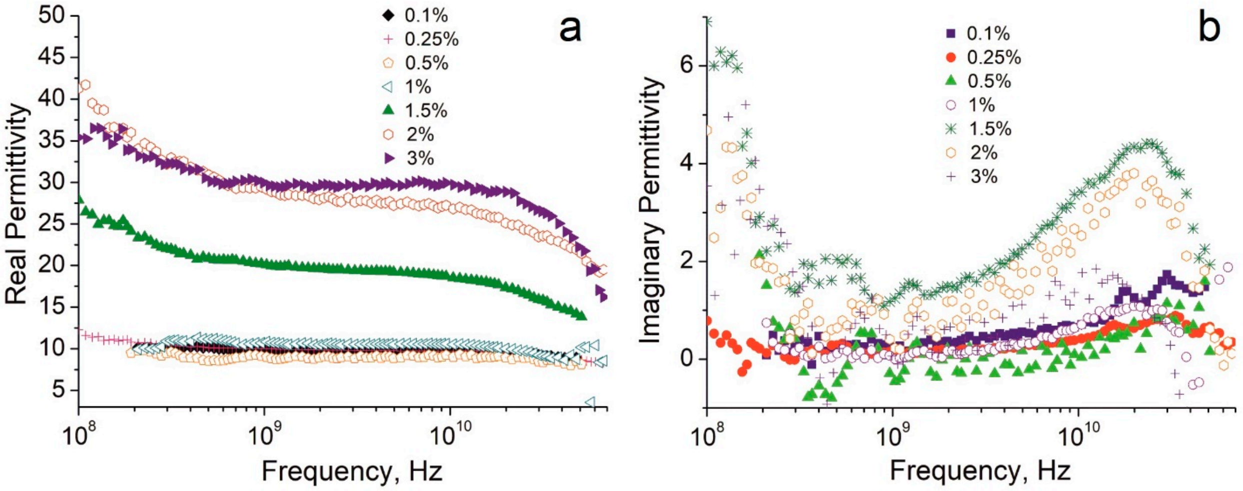

Figure S3. Frequency dependences of the real (a) and imaginary (b) parts of the complex permittivity for composites with different filler contents in the ultra-high frequency region.
